# Genetic diversity and drug susceptibility profiles of *Mycobacterium tuberculosis* obtained from Saint Peter’s TB specialized Hospital, Ethiopia

**DOI:** 10.1371/journal.pone.0218545

**Published:** 2019-06-24

**Authors:** Delesa Damena, Samuel Tolosa, Milkessa Hailemariam, Aboma Zewude, Adane Worku, Biruk Mekonnen, Temesgen Mohammed, Addisu Admasu, Emile R. Chimusa, Adane Mihret, Tamrat Abebe, Gobena Ameni

**Affiliations:** 1 Department of Microbiology and Parasitology, School of Medicine, College of Health Science Addis Ababa University, Addis Ababa, Ethiopia; 2 Aklilu Lemma Institute of Pathobiology, College of Health Sciences, Addis Ababa, Ethiopia; 3 Division of Human Genetics, Department of Pathology, Institute of Infectious Disease and Molecular Medicine, University Cape Town, Cape Town, South Africa; 4 Saint Peter’s TB specialized Hospital, Addis Ababa, Ethiopia; 5 Armauer Hansen Research Institute, Addis Ababa, Ethiopia; Jamia Hamdard, INDIA

## Abstract

**Background:**

Tuberculosis (TB) is one of the major public health problems in Ethiopia. Data on genetic diversity and resistance profile of circulating TB strains is critical for informing the national TB control program.

**Methods:**

A cross-sectional study was conducted on 213 smear positive pulmonary TB patients between 2015 and 2016. Sputum samples were cultured on LJ media following the Petroff’s method. Region of difference-9 (RD9)-deletion typing and spoligo-typing were performed for molecular analysis of *M*. *tuberculosis* at species and strain levels, respectively. Drug sensitivity and mutation patterns of the isolates were assessed by the conventional indirect proportion method and molecular line probe assays (LPAs), respectively. Data were analyzed using statistical package for social sciences (SPSS) software version 20.

**Results:**

Spoligo-typing of 150 *M*. *tuberculosis* isolates led to 57 different patterns of which 25 were new strains. The majority (71.6%) of the isolates were grouped in to 17 clusters consisting 2 to 24 isolates. The majority of the strains belonged to Euro-American lineage and the predominant spoligotypes were SIT 37 and SIT 149. MDR-TB was detected in 5.2% and 20.3% of new and retreatment cases, respectively. Two MDR-TB isolates exhibited additional resistance to one of the second line anti-TB drugs. Common gene mutations including *S531L*, *S315T1* and *M306V* were detected in RIF, INH and EMB resistant strains, respectively.

**Conclusions:**

The identification of several new strains, higher proportion of MDR-TB and higher clustering rate in this study, warrants the need for re-enforcement of the national TB control program. The detection of common gene mutations in the majority drug resistant strains might suggest the feasibility of LPAs for rapid screening of drug resistant *M*. *tuberculosis* strains in Ethiopia.

## Introduction

Ethiopia is among the top 30 TB burden countries in the world. TB and MDR/RR-TB incidence rate in the country is estimated to be 164 and 5.5 per 100 000 populations, respectively [1]. Thus, knowledge on molecular epidemiology and drug sensitivity profile of circulating *M*. *tuberculosis* strains is of utmost importance to inform the national TB control programs [2]. A number of drug sensitivity [3–6] and genetic diversity studies [7–9] were conducted in different parts of Ethiopia. However, the majority of these studies were limited to the investigations of either drug sensitivity profiles or genetic diversity and thus, unable to explain the complex inter-play between different factors that contribute to the spread of *M*. *tuberculosis* and its drug resistant forms. Worsening the problem, studies are lacking at referral hospital settings which may better represent the national picture.

Here, we present results of a comprehensive study on genetic diversity, drug sensitivity profile and mutation patterns of *M*. *tuberculosis* strains isolated from pulmonary tuberculosis cases at Saint Peter TB specialized Hospital.

## Methods

### Study design

A cross-sectional study was conducted between 2015 and 2016 at St. Peter’s TB specialized Hospital, a national reference center for TB diagnosis and treatment located in Addis Ababa. At the time of study, the Hospital had close to 200 beds and provide care for seriously ill TB patients and those with treatment-related complications. Thus, the *M*. *tuberculosis* isolates obtained from this Hospital might represent the majority of the strains circulating in the country. Smear positive pulmonary tuberculosis patients who were willing to give their informed consents were enrolled. Patients who were below 18 years old and those who started a treatment or retreatment regimen before one month at the time of the study were excluded. Each patient who met the inclusion criteria was assigned a serial identification number and important epidemiological and clinical data were collected.

### Sampling and mycobacterium culture

The sample size was determined by a formula recommended by WHO for surveillance of drug resistant TB [10]: Taking the prevalence of 1.6% from previous report [11], and 300 total number of new smear positive cases registered during one year in the area, 95% significance level, 1% margin of error, the required sample size was 202. However, 213 samples were collected to increase the precision of the study. Prior to sputum collection, structured questionnaire was administered to patients in order to obtain information on previous TB episode, socio-demographic characteristics, health conditions and life styles.

New cases and retreatment cases were defined according to the WHO guideline [10, 11]. Three sputum samples (spot-morning-spot) were collected and AFB was conducted at the St. Peter’s TB specialized Hospital according to the national TB and Leprosy control program guideline[11]. Positive sputa were stored in cold chain and transported to Aklilu Lema Institute of Pathobiology (ALIP) TB laboratories within 1 day. The specimens were cultured on LJ media following the Petroff’s method as outlined in [10]. Inspection of media was done every week for growth until 8 weeks. Bacterial colonies were examined using Ziehl-Neelsen staining method and AFB-positive isolates were collected in separate vials for DST and molecular analyses.

### Region of Difference-9 (RD-9) deletion typing and Spoligo-typing

RD-9 deletion typing was performed using RD-9 FlankFW, RD-9 Int and RD-9 FlankRev primers to discriminate *M*. *tuberculosis* from other MTB complex [12]. H37Rv and *M*. *bovis* 2122/97 was used as a positive control for *M*. *tuberculosis* and *M*. *bovis*, respectively while distilled water was used as a negative control. A band size of 396 bp and 575 bp was designated as *M*. *tuberculosis* and *M*. *bovis* or *M*. *africanum*, respectively. Spoligo-typing was performed as outlined in Kamerbeek *et al* [13] using a standard kit (Ocimum Biosolutions, Ijsselstein, The Netherlands). Hybridization patterns obtained from the reaction were converted into binary and octal formats. SIT number of the strains were then retrieved from the fourth international spoligotyping (SPolDB4 database) [14]. Major lineages and sub lineages were predicted using conformal Bayesian network (CBN) and knowledge-based Bayesian network (KBBN) analysis, respectively[14]. Spolgo-patterns that have not been registered prior were considered as orphan strains. Two or more isolates sharing identical spoligotype patterns were identified as clusters while those with single spoligo-pattern were considered as unique strains.

### Drug susceptibility testing (DST) and Line-probe assays

Conventional drug susceptibility testing (DST) was performed for the first line anti-TB drugs including INH (Isoniazid), Rifampicin (RIF), Ethambutol (EMB) and Streptomycin (STM) by the indirect proportion method on the enriched Middlebrook 7H10 agar media using a 24 well plate (Becton Dickinson Company, USA) as per standard operating procedure of Aklilu Lema Institute of Pathobiology TB laboratories adopted from [15,16]. A critical concentration of 0.2 μg/ml, 1.0 μg/ml, 5.0 μg/ml and 2.0 μg/ml was used for INH, RIF, EMB and STM, respectively as recommended elsewhere [16]. GenoType MTBDRplus version 2.0 and GenoType MTBDR*sl* version 1.0 (Hain Lifescience, Nehren, Germany) assays were performed as per manufacturer’s instruction [17].

### Data management and statistical analysis

All data were entered, organized and analyzed using the SPSS statistical software packages, version 20 (SPSS Inc., Chicago, IL, USA). Chi-square and logistic regression tests were applied for association analysis. Results were considered statistically significant at p < 0.05.

### Ethical approval

Ethical clearance was obtained from Institutional Review Board of College of Health Science department of Microbiology, Parasitology and Immunology and Ethical committee of St. Peter’s TB specialized Hospital before commencement of the actual activities. Informed consents were obtained from all the study participants.

## Results

### Socio-demographic characteristics of the study participants

Socio-demographic, health conditions and life styles of the study participants were presented in Table 1. A total of 213 of smear positive pulmonary tuberculosis patients consisting of 111 (52.1%) males and 102 (47.9%) females were participated. The mean and median age of the participants was 29 and 33, respectively. More than half (54.5%) of the patients were young (25–34 years old). Among the participants, 24.4% had history of contacts with TB patients. HIV status was known for 111 participants out of which 27% were positive. The majority of the participants came from Addis Ababa (46%) and Oromia (28.6%) regions and had a wide range of occupations including farmers (12.7%), employed (44.1%), daily laborers (3.3%), house wife (12.7%) and others (27.2%).

**Table 1:** Socio-demographic characteristics of smear positive TB patients at St. Peter’s TB specialized Hospital in 2015–2016, Addis Ababa, Ethiopia.

Variable	Frequency (n)	Percentage (%)
**Sex**		
Male	111	52.1
Female	102	49.9
**Age group**		
15–24	36	16.9
25–34	116	54.5
35–44	35	4.7
45–54	10	2.3
55–64	5	5.2
>64	11	16.4
**Region**		
Addis Ababa	98	46
Oromia	61	28.6
Amhara	32	15
SNNP	20	9.4
Tigray	2	0.9
**Residence**		
Urban	102	47.9
Rural	111	52.1
Ethnicity		
Oromo	85	40
Amhara	64	30
Tigre	23	11
Gurage	11	5
Others	30	14
**Educational level**		
Illiterate	82	38.5
Write and read	104	48.8
Higher education	27	12.7
**History of treatment**		
New cases	98	46
Retreatment cases	115	54
**HIV-status**		
HIV-positive	30	14.1
HIV-negative	71	33.3
Unknown	112	52
**History of contact with TB patients**		
Yes	52	24.4
No	161	75.6

### Genetic characteristics of *M*. *tuberculosis* isolates

Out of the 213 sputum samples, 151, 53 and 9 were culture positive, culture negative and contaminated, respectively. Almost all (150/151) culture positive isolates were identified as *M*. *tuberculosis* by RD-9 deletion typing. Spoligo-typing of these isolates (n = 150) led to 57 different patterns which correspond to a 38% genetic diversity. Of these, 32 patterns had been previously registered in the spoligotype databases (S1 Table) and the remaining patterns were orphans (S2 Table). The majority (73.3%) of the isolates were grouped in to 17 clusters consisting of 2 to 24 isolates. Binary logistic regression was applied to assess association of the clustering rate with socio-demographic data and drug resistance patterns (S3 Table). However, no significant associations were detected.

According to TB insight classification, the majority (74%) of the isolates belonged to Euro-American lineage followed by Indo-Oceanic (9.3%) and East-African-Indian (8.7%). Three isolates (2.1%) had patterns that do not correspond to any of the major lineages described in the databases and thus, designated as “unknown” (S1 and S2 Tables). Further classifications, revealed that the majority (69.3%, n = 104) of the isolates belonged to T family among which 51.9% (n = 54) and 26.9% (n = 28) was T1 and T3 sub-family, respectively. The second dominant family was CAS family (17.3%, n = 26) followed by Manu family (6.7%, n = 10). The remaining 1.3% (n = 2), 2.7% (n = 4) and 2.7% (n = 4) belonged to Haarlem1, LAM and EA14 families, respectively. The most dominant spoligotypes were SIT 37 and SIT 149 consisting of 24 and 20 isolates, respectively.

### Drug resistance profiles of *M*. *tuberculosis* isolates

Out of a total of 150 isolates, 13.3% (n = 20) demonstrated resistance to at least one of the first line anti-TB drugs (INH, RIF, STM and EMB) (Table 2). Four isolates, two each from new and retreatment cases exhibited INH monoresistance. MDR-TB was identified in 5.2% and 20.3% of the isolates obtained from new and retreatment cases, respectively. Combined resistance to only three drugs and only four drugs was observed in 10 and 4 isolates, respectively. Running GenoType MTBDRplus assay for all isolates showed a perfect match with DST in detecting INH and/or RIF (S4 Table). All the twenty drug resistant isolates were further retested by GenoType MTBDR*sl* to assess resistance to second line anti-TB drugs such as: fluoroquinolones (FLQ) and second-line injectable (SLID) drugs including kanamycin (KM), amikacin (AM), and capreomycin (CAP). One of the MDR-TB strains showed resistance to SLID and another one isolate showed resistance to FLQ (S4 Table). None of the MDR isolates fulfilled the criteria for XDR. GenoType MTBDR*sl* detected the majority (85.7%, n = 12) of EMB resistant isolates.

**Table 2:** Drug resistance profile of *M*. *tuberculosis* obtained from smear positive pulmonary patients at St. Peter’s TB specialized Hospital in 2015–2016, Addis Ababa, Ethiopia.

Drug resistance pattern	New cases n = 96	retreatment cases n = 54
**Resistance to only one drug**		
Only INH	2(2.1%)	2(3.7%)
**Resistance to only two drugs**		
RIF + INH	1(1%)	1(1.9%)
**Resistance to only three drugs**		
RIF+INH+EMB	3(3.1%)	7(13%)
**Resistance to only four drugs**		
RIF+INH+STM+EMBL	1(1%)	1(1.9%)
RIF+INH+EMB+SLID	-	1(1.9%)
RIF+INH+EMB+FLQ	-	1(1.9%)

INH, Isoniazid; RIF, Rifampicin; EMB, Ethambutol; STM, Streptomycin; FLQ, Fluoroquinolones; SLID, Second-line Injectable anti-tuberculosis drugs

### Mutation patterns of drug resistant *M*. *tuberculosis* isolates

The majority (81.3%, n = 13) of RIF resistant isolates exhibited mutation at *S531L* of *rpoB* gene (S5 Table). One RIF resistant isolate showed mutation in *D516V* region of *rpoB* gene while another isolate demonstrated mutation in *H516Y* region of *rpoB* gene. All INH resistant isolates showed mutations at *S315T1* of *katG* gene. One of the MDR isolates demonstrated hetero-resistance mutation pattern in which all the wild type bands were simultaneously observed with mutant (*rpoBMUT3 and katG MUT1*) bands.

In one of the MDR isolates, a wild type, WT8 band, was missed without gain of corresponding band (*S531L*) in the mutation regions. Similarly, in 4 of the 12 EMB resistant MDR-strains detected by the GenoType MTBDR*sl*, *embB WT* band was absent without gain in the corresponding *MUT2B* band (*M306V)*. These isolates were designated as MDR-TB with rare (unknown) mutations for which respective probes were not included in the nitrocellulose strips of the LPAs. The remaining 8 EMB resistant isolates demonstrated mutations at position *M306V* (S5 Table).

### Distribution of drug resistance pattern among *M*. *tuberculosis* strains and risk factors

The majority (13/20) of the *M*. *tuberculosis* that demonstrated resistance to at least one of the tested drugs, belonged to EA lineages (n = 13); among which 69% (n = 9) were T-family (Table 3). The remaining isolates belonged to EAI (n = 4) and IO (n = 3) all of which were classified under CAS family. We further assessed association between risk factors including previous treatment history, HIV status, residence, TB lineages, age groups, education levels and history of previous contact with TB patients (Table 4). Previous treatment was significantly associated with drug resistance (p = 0.045).

**Table 3:** Distribution of *resistant M*. *tuberculosis* isolates with different lineages.

*M*. *tuberculosis* lineages	*M*. *tuberculosis* family	Drug resistance pattern					
		Only INH	Only RIF+INH	Only RIF+INH+ EMB	Only RIF+INH+ STM+EMB	Only RIF+INH+ EMBL+SLID	Only RIP+INH+ EMBL+FLQ
EA(n = 13)	T (n = 9)	2	1	3	2		1
	Manu1(n = 3)			2		1	
	Haarlem (n = 1)		1				
EAI(n = EAI(4)	CAS (n = 4)	1		3			
IO(n = 3)	CAS(n = 3)	1		2			

EA, Euro-American; EAI, East-African-Indian; IO, Indo-Oceanic; INH, Isoniazid; RIF, Rifampicin; EMB, Ethambutol; STM, Streptomycin

FLQ, Fluoroquinolones; SLID, Second-line Injectable anti-tuberculosis drugs; T, Tuscany; CAS, Central Asian

**Table 4:** Association between drug resistance and risk factors.

Variables	N	OR	95%CI	*P-value*
**Treatment group**				
New cases	7			
retreated cases	13	0.04	0.14–1.0	0.045
**HIV-status**				
HIV-positive	16			
HIV-negative	4	4.40	1.30–10.50	0.08
**Residence**				
Urban	9			
Rural	11	0.87	0.34–2.40	0.77
**Previous contact with TB patient**				
Yes	6			
No	14	28.0	3.063–9.65	0.20
***M***. ***tuberculosis* lineages**				
EA	13			
EAI	4			
IO	3	0.92	0.28–2.30	0.11
**Age group**				
15–24	7			
25–34	5			
35–44	2			
45–54	2			
55–64	1			
>64	-	2.1	0.57–1.14	0.24
**Educational level**				
Illiterate	8			
Write and read	9			
Higher education	3	3.2	0.67–2.84	0.30

EA, Euro-American; EAI, East-African-Indian; IO, Indo-Oceanic

## Discussions

In this study, we investigated the genetic diversity, drug resistance profiles and mutation patterns of *M*. *tuberculosis* strains isolated from pulmonary tuberculosis cases at Saint Peter’s TB specialized Hospital. The majority of the patients were in young productive age group (25–34 years old). The fact that this age group is a driving force of the economy of the Ethiopia, might imply that TB is exerting a considerable impact on the economy of country.

The high strain diversity (38%) observed in this study, is not unexpected in national referral Hospital settings where patients from wide range of areas across the country provided TB treatment and diagnostic services. However, the existence of high proportion of new strains suggests that the genetic diversity of *M*. *tuberculosis* strains in Ethiopia have not yet been fully understood and needs to studied further. Moreover, the higher clustering rate (71.6%) of *M*. *tuberculosis* recorded in this study, may suggest the recent transmission of *M*. *tuberculosis* strains in the population and thus, warrants the need for strengthening TB control programs in the country.

The predominance of EA and IO lineages, T and CAS families, and SIT 37 and 149 observed in this study, is consistent with previous reports from Ethiopia [7, 8]. While EA lineage might have been introduced to the Ethiopia during the Italian invasion [18, 19], IO lineage is believed to be originated from Ethiopia and distributed to other countries [18, 19]. T-family has been described in the international database as a spoligotype that is common in Ethiopia, Kenya and Libya [14]. CAS family was reported from neighbor countries including Tanzania, Uganda, Sudan and Kenya [20–23] and has been known as a predominant spoligotype in Middle East and Central Asia [23].

The relatively higher prevalence rate of MDR-TB in retreatment cases (20.3%) compared to new cases (5.2%) in this study, is consistent with the earlier studies in Ethiopia and elsewhere [24, 25]. The strong association of previous TB treatment with MDR-TB might be attributed to several factors including inappropriate chemotherapy regimens, inadequate or irregular drug supply, unsatisfactory patient or clinical compliance and lack of supervision of treatment regimen among others [26]. More importantly, the higher prevalence rate of MDR-TB strains with additional resistance to the second line ant-TB drugs in this study, warrants the urgent need for re-enforcement of the TB Control Program. It should be noted that the proportion of resistance to the second line ant-TB drugs might be higher since the sensitivity of GenoType MTBDR*sl* used in this study is very low [27] and thus, more powered studies are needed to assess the XDR-TB status in Ethiopia.

In the current study, we detected common gene mutations including *S531L*, *S315T1* and *M306V* in RIF, INH and EMB resistant strains, respectively. This is consistent with previous studies in Ethiopia [28, 29], Uganda [30] and India [31] in which similar mutation patterns were reported. This may strongly suggest, the feasibility of LPAs for rapid screening of drug resistant *M*. *tuberculosis* strains in Ethiopia. Rapid detection of TB and its drug resistance profile enables timely initiation of appropriate anti-TB therapy and thereby reduce mortalities and transmissions rate [10].

In this study, we identified one isolate with a hetero-resistance mutation pattern in which all the wild type bands were detected simultaneously with *rpoBMUT3* and *katG MUT1* mutation bands. Hetero-resistance represents a natural variation in the population of *M*. *tuberculosis* [32]. TB infection with a heterogeneous *M*. *tuberculosis* population can be caused by a re-infection event or by an evolutionary genetic variation of a single infection event [33]. Hetero-resistance cannot be determined using routine laboratory methods including culture and other molecular tests [17]. However, LPA trips contain both wild type and mutant probes of the genes known to be involved in resistance to a given drug which makes it possible to simultaneously detect both susceptible and resistant populations from a patient sample [17]. This enables effective clinical management of patients harbouring hetero-resistant *M*. *tuberculosis* population by explaining why a patient is not responding to a given treatment. For instance, continuing RIF treatment in a patient that harbours hetero-resistant *M*. *tuberculosis* in *rpoB* gene would be ineffective and can lead to development of resistance to other drugs. Rapid detection of hetero-resistance by LPAs can effectively address such problems by guiding proper drug regimen and thus, influence the clinical outcomes of the infections [17].

In the current study, rare mutation patterns were detected in RIF (n = 1) and EMB (n = 4) resistant isolates. Rare mutation patterns are characterized by absence of hybridization signals at one or more of the different WT reaction zones without the presence of corresponding MUT bands. This might be attributed to mutations in other genomic regions that were not included in LPAs [34, 35]. Other techniques such as DNA sequencing may resolve these cases.

## Limitations

Even though we provided important information on genetic diversity and drug resistance profile of *M*. *tuberculosis* in Ethiopia, this study had several limitations including 1) We didn’t perform conventional DST for PZA and second-line anti-TB drugs; 2) Methods used in this study, such as: spoligo-typing and LPAs have lower resolution compared to advanced molecular techniques such as DNA sequencing and thus, some information might have been missed 3) The study was limited to a referral Hospital and hence, might not be representative of the TB cases circulating within the study period in the country.

## Conclusions

The majority of *M*. *tuberculosis* isolated in this study, belonged to the predominant spoligotypes in Ethiopia. However, the existence of high proportion of new strains suggests that the genetic diversity of *M*. *tuberculosis* strains in Ethiopia has not been yet fully understood and needs to be studied further. The prevalence of drug resistant TB recorded in the present study was higher than the national data both in new and in previously treated cases. Moreover, some of MDR-TB strains exhibited additional resistance to the second line ant-TB drugs warranting the need for re-enforcement of the TB Control Program. In the current study, the majority of drug resistant isolates demonstrated common mutations; highlighting the feasibility of mutation-based molecular tools for rapid screening of drug resistant *M*. *tuberculosis* strains in Ethiopia. Taken together, we presented baseline information on population structure, drug resistance and mutation patterns of *M*. *tuberculosis* in a TB-referral Hospital in Ethiopia and highlighted the importance of further molecular epidemiological studies.

## Supporting information

S1 Table Shared Spoligotype patterns of *M.tuberculosis* isolates (n = 113) obtained from smear positive pulmonary patients at St. Peter’s TB specialized Hospital, in 2015–2016, Addis Ababa, Ethiopia. (PDF)

S2 Table Orphan Spoligotype patterns of *M. tuberculosis* isolates (n = 37) obtained from smear positive pulmonary patients at St. Peter’s TB specialized Hospital in 2015–2016, Addis Ababa, Ethiopia. (PDF)

S3 Table Association of clustered *M. tuberculosis* isolates with sociodemographic data and drug resistance pattern (n = 150). (PDF)

S4 Table Drug resistance profile of *M. tuberculosis* isolates obtained from smear positive pulmonary patients at St. Peter’s TB specialized Hospital in 2015–2016, Addis Ababa, Ethiopia determined by LPAs. (PDF)

S5 Table Mutation patterns of drug resistant *M. tuberculosis* isolates(n = 20) obtained from smear positive pulmonary patients at St. Peter’s TB specialized Hospital in 2015–2016, Addis Ababa, Ethiopia determined by GenoType MTBDRplus and MTBDR*sl* assays. (PDF)

## References

[1] World Health Organization. Global Tuberculosis Report. WHO, Geneva Switzerland 2018 http://www.who.int/tb/data

[2] Lazzarini LCO, Huard RC, Boechat NL, Gomes HM, Oelemann MC, Kurepina N, et al Discovery of a novel Mycobacterium tuberculosis lineage that is a major cause of tuberculosis in Rio de Janeiro, Brazil. J Clin Microbiol. 2007;45: 3891–3902. 10.1128/JCM.01394-07 17898156 PMC2168543

[3] Bruchfeld J, Aderaye G, Palme IB, Bjorvatn B, Ghebremichael S, Hoffner S, et al Evaluation of outpatients with suspected pulmonary tuberculosis in a high HIV prevalence setting in Ethiopia: clinical, diagnostic and epidemiological characteristics. Scand J Infect Dis. 2002;34:331–337 12069014 10.1080/00365540110080025

[4] Eyob G, Guebrexabher H, Lemma E, Wolday D, Gebeyehu M, Abated G, et al Drug susceptibility of Mycobacterium tuberculosis in HIV-infected and -uninfected Ethiopians and its impact on outcome after 24 months of follow-up. Int J Tuberc Lung Dis. 2004;8: 1388–1391. 10.1080/00016480600818054 15581212

[5] Yimer SA, Agonafir M, Derese Y, Sani Y, Bjune GA, Holm-Hansen C. Primary drug resistance to anti-tuberculosis drugs in major towns of Amhara region, Ethiopia. APMIS. 2012;120: 503–509. 10.1111/j.1600-0463.2011.02861.x 22583363

[6] Tessema B, Beer J, Emmrich F, Sack U, Rodloff AC. First and second line anti-tuberculosis drug resistance in Northwest Ethiopia. Int J Tuberc Lung Dis. 2012, 16:805–811. 10.5588/ijtld.11.0522 22390880

[7] Mihret A, Bekele Y, Loxton AG, Jordan AM, Yamuah L, Aseffa A, et al Diversity of Mycobacterium tuberculosis Isolates from New Pulmonary Tuberculosis Cases in Addis Ababa, Ethiopia. Tuberc Res Treat. 2012;2012: 892079 10.1155/2012/892079 23227330 PMC3513727

[8] Diriba B, Berkessa T, Mamo G, Tedla Y, Ameni G. Spoligotyping of multidrug-resistant Mycobacterium tuberculosis isolates in Ethiopia. Int J Tuberc Lung Dis. 2013;17: 246–250. 10.5588/ijtld.12.0195 23317962

[9] Mihret A, Bekele Y, Aytenew M, Assefa Y, Abebe M, Wassie L, et al Modern lineages of Mycobacterium tuberculosis in Addis Ababa, Ethiopia: Implications for the tuberculosis control programe. Afr Health Sci. 2012;12: 339–344. 10.4314/ahs.v12i3.15 23382750 PMC3557690

[10] World Health Organization. Guidelines for surveillance of drug resistance in tuberculosis. WHO, Geneva Switzerland 2013

[11] Ministry of Health of Ethiopia. Tuberculosis, Leprosy and TB/HIV Prevention and Control Program Manual, Addis Ababa, Ethiopia 2013

[12] Huard RC, Lazzarini LC de O, Butler WR, van Soolingen D, Ho JL. PCR-based method to differentiate the subspecies of the Mycobacterium tuberculosis complex on the basis of genomic deletions. J Clin Microbiol. 2003;41: 1637–1650. 10.1128/JCM.41.4.1637-1650.2003 12682155 PMC153936

[13] Kamerbeek J, Schouls LEO, Kolk A, Kuijper S, Bunschoten A, Molhuizen H, et al Simultaneous Detection and Strain Differentiation of Mycobacterium tuberculosis for diagnosis and Epidemiology. J Clin Microbiol. 1997; 35 907–14. 9157152 10.1128/jcm.35.4.907-914.1997 PMC229700

[14] Brudey K, Driscoll JR, Rigouts L, Prodinger WM, Gori A, Al-Hajoj SA, et al Mycobacterium tuberculosis complex genetic diversity: Mining the fourth international spoligotyping database (SpolDB4) for classification, population genetics and epidemiology. BMC Microbiol. 2006;6: 1–17. 10.1186/1471-2180-6-1 16519816 PMC1468417

[15] Wedajo W, Schön T, Bedru A, Kiros T, Hailu E, Mebrahtu T, et al A 24-well plate assay for simultaneous testing of first and second line drugs against Mycobacterium tuberculosis in a high endemic setting. BMC Res Notes. 2014;7: 1–8. 10.1186/1756-0500-7-1 25108648 PMC4267144

[16] Van Klingeren B, Dessens-Kroon M, Van Der Laan T, Kremer K, Van Soolingen D. Drug susceptibility testing of Mycobacterium tuberculosis complex by use of a high-throughput, reproducible, absolute concentration method. J Clin Microbiol. 2007;45: 2662–2668. 10.1128/JCM.00244-07 17537932 PMC1951260

[17] Barnard M, Parsons L, Miotto P, Cirillo D, Feldmann K, Gutierrez C, et al Molecular Detection of Drug-Resistant Tuberculosis By Line Probe Assay. UNITAID- Expand Proj. 2012;

[18] Garedew L, Mihret A, Mamo G, Abebe T, Firdessa R, Bekele Y, et al Strain diversity of mycobacteria isolated from pulmonary tuberculosis patients at Debre Birhan Hospital, Ethiopia. Int J Tuberc Lung Dis. 2013;17: 1076–1081. 10.5588/ijtld.12.0854 23827032

[19] Belay M, Ameni A, Bjune G, Couvin D, 3 Rastogi N, et al Strain diversity of Mycobacterium tuberculosis isolates from pulmonary tuberculosis patients in Afar pastoral region of Ethiopia. Biomed Res Int. 2014;2014: 238532 10.1155/2014/238532 24734230 PMC3966356

[20] Sharaf Eldin GS, Fadl-Elmula I, Ali MS, Ali AB, Salih ALGA, Mallard K, et al Tuberculosis in Sudan: A study of Mycobacterium tuberculosis strain genotype and susceptibility to anti-tuberculosis drugs. BMC Infect Dis. BioMed Central Ltd; 2011;11: 219 10.1186/1471-2334-11-219 21846389 PMC3166935

[21] Githui WA, Jordaan AM, Juma ES, Kinyanjui P, Karimi FG, Kimwomi J, et al Identification of MDR-TB Beijing/W and other Mycobacterium tuberculosis genotypes in Nairobi, Kenya. Int J Tuberc Lung Dis. 2004;8: 352–360. 15139475

[22] Ogaro TD, Githui W, Kikuvi G, Okari J, Asiko V, Wangui E, et al Diversity of Mycobacterium tuberculosis strains in Nairobi, Kenya. Afr J Health Sci. 2012;20: 82–90.

[23] Bhanu NV, Van Soolingen D, Van Embden JDA, Dar L, Pandey RM, Seth P. Predominace of a novel Mycobacterium tuberculosis genotype in the Delhi region of India. Tuberculosis. 2002;82: 105–112. 10.1054/tube.2002.0332 12356462

[24] Selamawit H, Girmay M, Belaineh G, Muluken M, Alemayehu M, Suarez P, et al Determinants of multidrug-resistant tuberculosis in patients who underwent first-line treatment in Addis Ababa: a case control study. BMC Public Health. 2013;13: 1–9. 10.1186/1471-2458-13-1 23981845 PMC4015150

[25] Agonafir M, Lemma E, Wolde-Meskel D, Goshu S, Santhanam A. Phenotypic and genotypic analysis of multidrug-resistant tuberculosis in Ethiopia. Int J Tuberc Lung Dis. 2010;14:1259–1265. 20843416

[26] Faustini A, Hall AJ, Perucci CA. Risk factors for multidrug resistant tuberculosis in Europe: A systematic review. Thorax. 2006;61: 158–163. 10.1136/thx.2005.045963 16254056 PMC2104570

[27] World Health Organization. Use of molecular Line Probe Assay for the detection of resistance to second-line anti-tuberculosis drugs. Expert group meeting report, Geneva 2013; 1–52.

[28] Bedewi Omer Z, Mekonnen Y, Worku A, Zewde A, Medhin G, Mohammed T, et al Evaluation of the GenoType MTBDRplus assay for detection of rifampicin- and isoniazid-resistant Mycobacterium tuberculosis isolates in central Ethiopia. Int J Mycobacteriol. 2016;5: 475–481. 10.1016/j.ijmyco.2016.06.005 27931690

[29] Biadglegne F, Tessema B, Rodloff AC, Sack U. Magnitude of gene mutations conferring drug resistance in Mycobacterium tuberculosis isolates from lymph node aspirates in ethiopia. Int J Med Sci. 2013;10: 1589–1594. 10.7150/ijms.6806 24046537 PMC3775120

[30] Albert H, Bwanga F, Mukkada S, Nyesiga B, Ademun JP, Lukyamuzi G et al Rapid screening of MDR-TB using molecular Line Probe Assay is feasible in Uganda. BMC Infect Dis. 2010;10: 41 10.1186/1471-2334-10-41 20187922 PMC2841659

[31] Yadav RN, Singh BK, Sharma SK, Sharma R, Soneja M, Sreenivas V, et al Comparative Evaluation of GenoType MTBDRplus Line Probe Assay with Solid Culture Method in Early Diagnosis of Multidrug Resistant Tuberculosis (MDR-TB) at a Tertiary Care Centre in India. PLoS One. 2013;8: 1–6. 10.1371/journal.pone.0072036 24039735 PMC3764192

[32] Andrews JR, Gandhi NR, Moodley P, Shah NS, Bohlken L, Moll AP, et al Exogenous Reinfection as a Cause of Multidrug‐Resistant and Extensively Drug‐Resistant Tuberculosis in Rural South Africa. J Infect Dis. 2008;198: 1582–1589. 10.1086/592991 18847372

[33] Small PM, Shafer RW, Hopewell PC, Singh SP, Murphy MJ, Desmond E et al Exogenous reinfection with multidrug-resistant Mycobacterium tuberculosis in patients with advanced HIV infection. N Engl J Med. 1993: 1137–1144. 10.1056/NEJM199304223281601 8096066

[34] Van Deun A, Barrera L, Bastian I, Fattorini L, Hoffmann H, Kam KM, et al Mycobacterium tuberculosis strains with highly discordant rifampin susceptibility test results. J Clin Microbiol. 2009;47: 3501–3506. 10.1128/JCM.01209-09 19759221 PMC2772627

[35] Cuevas-Córdoba B, Juárez-Eusebio DM, Almaraz-Velasco R, Muñiz-Salazar R, Laniado-Laborin R, Zenteno-Cuevas R. Mutation at embB codon 306, a potential marker for the identification of multidrug resistance associated with ethambutol in Mycobacterium tuberculosis. Antimicrob Agents Chemother. 2015;59: 5455–5462. 10.1128/AAC.00117-15 26124153 PMC4538480

