# Supplementary material for: Genetic diversity and drug susceptibility profiles of Mycobacterium tuberculosis obtained from Saint Peter’s TB specialized Hospital, Ethiopia
Source: PLoS One. 2019 Jun 24;14(6):e0218545. doi: 10.1371/journal.pone.0218545 (PMC6590806; doi:10.1371/journal.pone.0218545)
Supplement: S1 Table — (PDF) [file pone.0218545.s001.pdf]

S1 Table. Shared Spoligotype patterns of *M. tuberculosis* isolates (n=113) obtained from smear positive pulmonary patients at St. Peter's TB specialized Hospital in 2015 - 2016, Addis Ababa, Ethiopia

SN	SIT	Isolates (n)	Family	CBN Lineage/sublineage	Octal number	Binary format
1	1426	4	T1	EA	777377777607111	
2	52	3	T2	EA	777777777760731	
3	25	9	CAS-Delhi	EAI	703777740003171	
4	102	1	T	EA	777703777760771	
5	3900	6	Manu2	EA	777347777763771	
6	1634	2	Manu2	EA	777777777723771	
7	1821	14	T	EA	777347777760771	
8	61	1	LAM10	EA	777777743760771	
9	44	1	T1	EA	777777757760771	
10	50	1	T1	EA	777777777720771	
11	241	1	T1	EA	777777777760411	
12	804	1	T1	EA	477777777760771	
13	1264	1	CAS-Delhi	EAI	703777740000000	
14	2820	2	T2	EA	777777767760731	
15	584	1	T2	EA	777775777760731	
16	37	24	T1	EA	777737777760771	
17	54	1	Manu2	EA	777777777763771	
18	462	1	T1	EA	777777777560771	
19	53	6	T1	EA	777777777760771	
20	358	1	T1	EA	716777777770771	
21	247	1	CAS	UN	703777740003471	
22	121	1	T1	EA	777777775720771	
23	777	1	T1	EA	777777777420771	
24	149	20	T3-ETH	EA	777000377760771	
25	1675	1	CAS	UN	703367400001771	
26	100	1	Manu1	EA	777777777773771	
27	241	2	T1	EA	777777777760411	
28	3898	1	CAS	EAI2	677777077413771	
29	20	1	LAM1	EA	677777607760771	
30	2693	1	CAS-Delhi	EAI	703763740003771	
31	205	1	T	EA	737777777760771	
32	26	1	CAS-Delhi	EAI	703777740003771	

CAS, Central Asian; LAM, Latin American Mediterranean; EAI, East African Indian; H, Haarlem; T, Tuscany; UN, Unclassified; SIT, Spoligo International Type. CBN: Conformal Bayesian Network. The black and white boxes show the presence and absence of the specific at positions 1-43 in the DRlocus.
