# Supplementary material for: Genetic diversity and drug susceptibility profiles of Mycobacterium tuberculosis obtained from Saint Peter’s TB specialized Hospital, Ethiopia
Source: PLoS One. 2019 Jun 24;14(6):e0218545. doi: 10.1371/journal.pone.0218545 (PMC6590806; doi:10.1371/journal.pone.0218545)
Supplement: S2 Table — (PDF) [file pone.0218545.s002.pdf]

S2 Table. Orphan Spoligotype patterns of *M. tuberculosis* isolates (n=37) obtained from smear positive pulmonary patients at St. Peter's TB specialized Hospital in 2015-2016, Addis Ababa, Ethiopia

S/N	Isolates(n)	Family	CBN lineage/sublineage	Octal number	Binary format
1	2	T1	EA	777777575720771	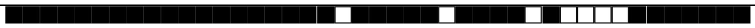
2	1	LAM1	EA	677777407740771	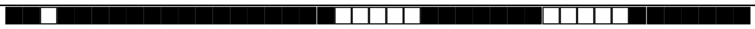
3	1	T1	EA	777777577420771	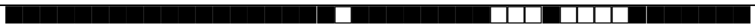
4	1	T3	EA	777000177740771	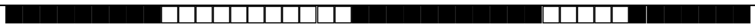
5	1	CAS	UN	703777500003771	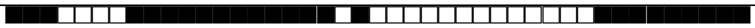
6	1	T3	EA	777000177760771	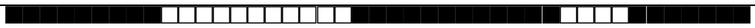
7	1	EA14	IO	763777540003171	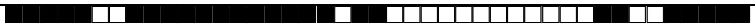
8	1	EA14	IO	777777540003771	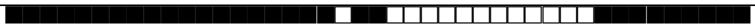
9	3	T1	EA	777737577760771	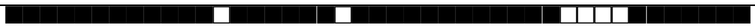
10	1	EA14	IO	773777540003171	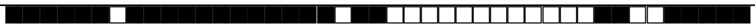
11	1	EA14	IO	747777540005171	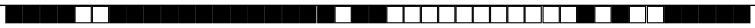
12	1	T3	EA	777001777607771	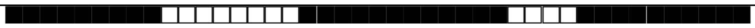
13	1	T3	EA	777000177760771	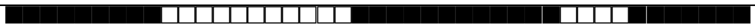
14	1	T1	EA	777727577760731	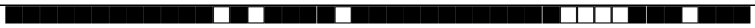
15	1	CAS	IO	723377400001771	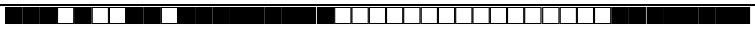
16	1	LAM9	EA	777777401760771	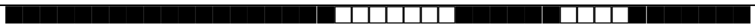
17	2	CAS	IO	723777740003571	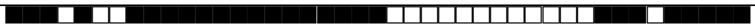
18	1	T1	EA	777777647760771	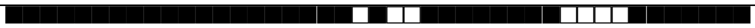
19	3	CAS	IO	723377400001771	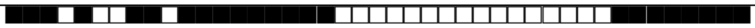
20	2	T1	EA	677777647760771	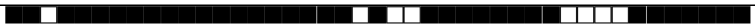
21	1	T3	EA	777002377760771	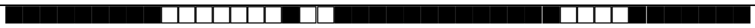
22	4	CAS	IO	723777740003571	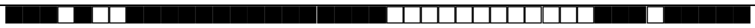
23	1	T3	EA	777003377760771	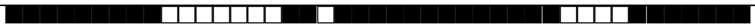
24	2	T3	EA	777003377760771	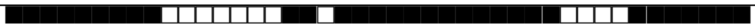
25	2	Haarlem	EA	777430477207711	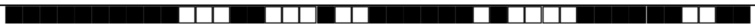

CAS, Central Asian; LAM, Latin American Mediterranean; EAI, East African Indian; H, Haarlem; T, Tuscany; UN, Unclassified; SIT, Spoligo International Type. CBN: Conformal Bayesian Network. The black and white boxes show the presence and absence of the specific at positions 1-43 in the DRlocus.
