# Supplementary material for: Genetic diversity and drug susceptibility profiles of Mycobacterium tuberculosis obtained from Saint Peter’s TB specialized Hospital, Ethiopia
Source: PLoS One. 2019 Jun 24;14(6):e0218545. doi: 10.1371/journal.pone.0218545 (PMC6590806; doi:10.1371/journal.pone.0218545)
Supplement: S3 Table — (PDF) [file pone.0218545.s003.pdf]

S3 Table. Association of clustered *M. tuberculosis* isolates with sociodemographic data and drug resistance pattern (n=150)

Variable	Clustered spoligotype	None clustered Spoligotype	P-value
History of treatment			
New cases	52	25	
Previously treated cases	49	24	0.26
HIV-status			
HIV-positive	12	11	
HIV-negative	23	25	
Unknown	38	41	0.21
Drug resistance			
Pan-Susceptible	86	44	
Any resistance to the tested drugs	14	6	0.1
Sex			0.88
Male	45	25	
Female	56	15	0.063
Age group			
15-24	16	7	
25–34	58	31	
35–44	14	7	
45–54	3	3	
55–64	2	1	
>64	6	1	0.75
Residence			
Urban	48	22	
Rural	53	18	0.46
Region			
Addis Ababa	36	29	
Oromia	30	20	
Amhara	10	7	
SNNP	11	6	
Tigria	1	0	0.72
Educational level			
Illiterate	44	22	
Write and read	47	19	
Higher education	10	8	0.22
