# Supplementary material for: Genetic diversity and drug susceptibility profiles of Mycobacterium tuberculosis obtained from Saint Peter’s TB specialized Hospital, Ethiopia
Source: PLoS One. 2019 Jun 24;14(6):e0218545. doi: 10.1371/journal.pone.0218545 (PMC6590806; doi:10.1371/journal.pone.0218545)
Supplement: S4 Table — (PDF) [file pone.0218545.s004.pdf]

S4 Table. Drug resistance profile of *M. tuberculosis* isolates obtained from smear positive pulmonary patients at St. Peter's TB specialized Hospital in 2015-2016, Addis Ababa, Ethiopia determined by LPAs

S/N	Drug resistance profile of *M. tuberculosis* isolates					Type of resistance
	GenoType MTBDRplus		GenoType MTBDR*sl*			
	INH	RIF	EMB	SLID	FLQ	
1	Negative	Negative	Negative	Negative	Negative	
2	Negative	Negative	Negative	Negative	Negative	
3	Negative	Negative	Negative	Negative	Negative	
4	Negative	Negative	Negative	Negative	Negative	
5	Negative	Negative	Negative	Negative	Negative	
6	Negative	Negative	Negative	Negative	Negative	
7	Negative	Negative	Negative	Negative	Negative	
8	Negative	Negative	Negative	Negative	Negative	
9	Positive	Negative	Negative	Negative	Negative	INH-mono-resistant
10	Negative	Negative	Negative	Negative	Negative	
11	Negative	Negative	Negative	Negative	Negative	
12	Negative	Negative	Negative	Negative	Negative	
13	Positive	positive	Positive	Negative	positive	MDR+FLQ
14	Negative	Negative	Negative	Negative	Negative	
15	Negative	Negative	Negative	Negative	Negative	
16	Positive	Positive	Positive	Negative	Negative	MDR
17	Negative	Negative	Negative	Negative	Negative	
18	Positive	Positive	Positive	Negative	Negative	MDR
19	Negative	Negative	Negative	Negative	Negative	
20	Negative	Negative	Negative	Negative	Negative	
21	Negative	Negative	Negative	Negative	Negative	
22	Negative	Negative	Negative	Negative	Negative	
23	Negative	Negative	Negative	Negative	Negative	
24	Negative	Negative	Negative	Negative	Negative	
25	Negative	Negative	Negative	Negative	Negative	
26	Negative	Negative	Negative	Negative	Negative	
27	Negative	Negative	Negative	Negative	Negative	
28	Negative	Negative	Negative	Negative	Negative	
29	Negative	Negative	Negative	Negative	Negative	
30	Negative	Negative	Negative	Negative	Negative	
31	Negative	Negative	Negative	Negative	Negative	
32	Negative	Negative	Negative	Negative	Negative	
33	Positive	Positive	Positive	Negative	Negative	MDR
34	Negative	Negative	Negative	Negative	Negative	
35	Negative	Negative	Negative	Negative	Negative	
36	Negative	Negative	Negative	Negative	Negative	

37	Negative	Negative	Negative	Negative	Negative	
38	Negative	Negative	Negative	Negative	Negative	
39	Positive	Positive	Positive	Negative	Negative	MDR
40	Negative	Negative	Negative	Negative	Negative	
41	Negative	Negative	Negative	Negative	Negative	
42	Positive	positive	Negative	Negative	Negative	MDR
43	Negative	Negative	Negative	Negative	Negative	
44	Negative	Negative	Negative	Negative	Negative	
45	Negative	Negative	Negative	Negative	Negative	
46	Negative	Negative	Negative	Negative	Negative	
47	Positive	Positive	Positive	Negative	Negative	MDR
48	Negative	Negative	Negative	Negative	Negative	
49	Negative	Negative	Negative	Negative	Negative	
50	Positive	positive	positive	Negative	Negative	MDR
51	Negative	Negative	Negative	Negative	Negative	
52	Negative	Negative	Negative	Negative	Negative	
53	Negative	Negative	Negative	Negative	Negative	
54	Negative	Negative	Negative	Negative	Negative	
55	Negative	Negative	Negative	Negative	Negative	
56	Negative	Negative	Negative	Negative	Negative	
57	Negative	Negative	Negative	Negative	Negative	
58	Negative	Negative	Negative	Negative	Negative	
59	Positive	positive	positive	Negative	Negative	MDR
60	Positive	Negative	Negative	Negative	Negative	INH-monoresistant
61	Negative	Negative	Negative	Negative	Negative	
62	Negative	Negative	Negative	Negative	Negative	
63	Negative	Negative	Negative	Negative	Negative	
64	Negative	Negative	Negative	Negative	Negative	
65	Negative	Negative	Negative	Negative	Negative	
66	Negative	Negative	Negative	Negative	Negative	
67	Negative	Negative	Negative	Negative	Negative	
68	Negative	Negative	Negative	Negative	Negative	
69	Negative	Negative	Negative	Negative	Negative	
70	Negative	Negative	Negative	Negative	Negative	
71	Negative	Negative	Negative	Negative	Negative	
72	Positive	Negative	Negative	Negative	Negative	INH-monoresistant
73	Negative	Negative	Negative	Negative	Negative	
74	Negative	Negative	Negative	Negative	Negative	
75	Negative	Negative	Negative	Negative	Negative	
76	Negative	Negative	Negative	Negative	Negative	
77	Negative	Negative	Negative	Negative	Negative	
78	Negative	Negative	Negative	Negative	Negative	
79	Negative	Negative	Negative	Negative	Negative	
80	Negative	Negative	Negative	Negative	Negative	
81	Negative	Negative	Negative	Negative	Negative	

82	Negative	Negative	Negative	Negative	Negative	
83	Negative	Negative	Negative	Negative	Negative	
84	Negative	Negative	Negative	Negative	Negative	
85	Negative	Negative	Negative	Negative	Negative	
86	Negative	Negative	Negative	Negative	Negative	
87	Negative	Negative	Negative	Negative	Negative	
88	Negative	Negative	Negative	Negative	Negative	
89	Negative	Negative	Negative	Negative	Negative	
90	Negative	Negative	Negative	Negative	Negative	
91	Negative	Negative	Negative	Negative	Negative	
92	Negative	Negative	Negative	Negative	Negative	
93	Negative	Negative	Negative	Negative	Negative	
94	Negative	Negative	Negative	Negative	Negative	
95	Negative	Negative	Negative	Negative	Negative	
96	Negative	Negative	Negative	Negative	Negative	
97	Negative	Negative	Negative	Negative	Negative	
98	Negative	Negative	Negative	Negative	Negative	
99	Negative	Negative	Negative	Negative	Negative	
100	Negative	Negative	Negative	Negative	Negative	
101	Negative	Negative	Negative	Negative	Negative	
102	Negative	Negative	Negative	Negative	Negative	
103	Negative	Negative	Negative	Negative	Negative	
104	Negative	Negative	Negative	Negative	Negative	
105	Positive	positive	positive	Negative	Negative	MDR
106	Negative	Negative	Negative	Negative	Negative	
107	Negative	Negative	Negative	Negative	Negative	
108	Negative	Negative	Negative	Negative	Negative	
109	Negative	Negative	Negative	Negative	Negative	
110	Negative	Negative	Negative	Negative	Negative	
111	Negative	Negative	Negative	Negative	Negative	
112	Positive	positive	positive	Negative	Negative	MDR
113	Negative	Negative	Negative	Negative	Negative	
114	Negative	Negative	Negative	Negative	Negative	
115	Negative	Negative	Negative	Negative	Negative	
116	Negative	Negative	Negative	Negative	Negative	
117	Negative	Negative	Negative	Negative	Negative	
118	Positive	positive	Negative	Negative	Negative	MDR
119	Negative	Negative	Negative	Negative	Negative	
120	Negative	Negative	Negative	Negative	Negative	
121	Negative	Negative	Negative	Negative	Negative	
122	Positive	positive	positive	Negative	Negative	MDR
123	Negative	Negative	Negative	Negative	Negative	
124	Negative	Negative	Negative	Negative	Negative	
125	Negative	Negative	Negative	Negative	Negative	
126	Negative	Negative	Negative	Negative	Negative	

127	Negative	Negative	Negative	Negative	Negative	
128	Negative	Negative	Negative	Negative	Negative	
129	Negative	Negative	Negative	Negative	Negative	
130	Negative	Negative	Negative	Negative	Negative	
131	Negative	Negative	Negative	Negative	Negative	
132	Positive	positive	Negative	Negative	Negative	MDR
133	Negative	Negative	Negative	Negative	Negative	
134	Negative	Negative	Negative	Negative	Negative	
135	Negative	Negative	Negative	Negative	Negative	
136	Negative	Negative	Negative	Negative	Negative	
137	Negative	Negative	Negative	Negative	Negative	
138	Positive	Negative	Negative	Negative	Negative	INH-mono-resistant
139	Negative	Negative	Negative	Negative	Negative	
140	Negative	Negative	Negative	Negative	Negative	
141	Negative	Negative	Negative	Negative	Negative	
142	Negative	Negative	Negative	Negative	Negative	
143	Positive	positive	Negative	Negative	Negative	MDR
144	Negative	Negative	Negative	Negative	Negative	
145	Negative	Negative	Negative	Negative	Negative	
146	Negative	Negative	Negative	Negative	Negative	
147	Negative	Negative	Negative	Negative	Negative	
148	Negative	Negative	Negative	Negative	Negative	
149	Positive	Positive	Positive	positive	Negative	MDR+SLID
150	Negative	Negative	Negative	Negative	Negative	
