# Supplementary material for: Genetic diversity and drug susceptibility profiles of Mycobacterium tuberculosis obtained from Saint Peter’s TB specialized Hospital, Ethiopia
Source: PLoS One. 2019 Jun 24;14(6):e0218545. doi: 10.1371/journal.pone.0218545 (PMC6590806; doi:10.1371/journal.pone.0218545)
Supplement: S5 Table — (PDF) [file pone.0218545.s005.pdf]

S5 Table. Mutation patterns of drug resistant *M. tuberculosis* isolates(n=20) obtained from smear positive pulmonary patients at St. Peter's TB specialized Hospital in 2015-2016, Addis Ababa, Ethiopia determined by GenoType MTBDRplus and MTBDR*sl* assays

S/N	INH				RIF		EMB		SLID		FLQ		*Type of M. tuberculosis resistance*
	KatG		*inhA*		*rpoB*		*embB*		*Rrs*		*gyrA*		
	WT-missed	MUT-gained	WT-missed	MUT-gained	WT-missed	MUT-gained	WT-missed	MUT-gained	WT-missed	MUT-gained	WT-missed	MUT-gained	
1	WT1	*MUT2 (S315T1)*	-	-	-	-	-	-	-	-	-	-	INH-mono-resistant
2	WT1	*MUT2 (S315T1)*	-	-	WT8	*MUT3 (S531L)*	*WT1*	*MUT2B (M306V)*	-	-	*gyrAWT3*	*MUT1 (A90V)*	MDR+FLQ
3	WT1	*MUT2 (S315T1)*	-	-	WT8	*MUT3 (S531L)*	*WT1*	-	-	-	-	-	MDR-rare mutation pattern for EMB
4	WT1	*MUT2 (S315T1)*	-	-	WT8	*MUT3 (S531L)*	*WT1*	*MUT2B (M306V)*	-	-	-	-	MDR
5	WT1	*MUT2 (S315T1)*	-	-	WT8	*MUT3 (S531L)*	*WT1*	-	-	-	-	-	MDR-rare mutation pattern for EMB
6	WT1	*MUT2 (S315T1)*	-	-	WT7	*MUT2B (H516D)*	*WT1*	*MUT2B (M306V)*	-	-	-	-	MDR
7	-	*MUT2 (S315T1)*	-	-	-	*MUT2B (H516D_*	-	-	-	-	-	-	MDR-Hetero-resistant
8	WT1	*MUT2 (S315T1)*	-	-	WT8	*MUT3 (S531L)*	*WT1*	-	-	-	-	-	MDR-rare mutation pattern for EMB
9	WT1	*MUT2 (S315T1)*	-	-	WT8	*MUT3 (S531L)*	*WT1*	-	-	-	-	-	MDR-rare mutation pattern for EMB
10	WT1	*MUT2 (S315T1)*	-	-	WT8	*MUT3 (S531L)*	*WT1*	*MUT2B (M306V)*	-	-	-	-	MDR
11	WT1	*MUT1 (S315T1)*	-	-	-	-	-	-	-	-	-	-	INH-mono-resistant
12	WT1	*MUT1 (S315T1)*	-	-	-	-	-	-	-	-	-	-	INH-mono-resistant
13	WT1	*MUT2 (S315T1)*	-	-	WT8	*MUT3 (S531L)*	*WT1*	*MUT2B (M306V)*	-	-	-	-	MDR
14	WT1	*MUT2 (S315T1)*	-	-	WT7	*MUT2B H516D*	*WT1*	*MUT2B (M306V)*	-	-	-	-	MDR

15	WT1	*MUT2 (S315T1)*	-	-	WT8	*MUT3 (S531L)*	-	-	-	-	-	-	MDR
16	WT1	*MUT2 (S315T1)*	-	-	WT8	*MUT3 (S531L)*	*WT1*	*MUT2B (M306V)*	-	-	-	-	MDR
17	WT1	*MUT2 (S315T1)*	-	-	WT8	*MUT3 (S531L)*	-	-	-	-	-	-	MDR
18	WT1	*MUT2 (S315T1)*	-	-	-	-	-	-	-	-	-	-	INH-mono-resistant
19	WT1	*MUT2 (S315T1)*	-	-	WT8	-	-	-	-	-	-	-	MDR-rare mutation pattern for RIF
20	WT1	*MUT2 (S315T1)*	-	-	WT8	*MUT3 (S531L)*	*WT1*	*MUT2B (M306V)*	*WT1*	*MUT1 (A1401G)*	-	-	MDR+SLID

INH, Isoniazid; RIF, Rifampicin; EMB, Ethambutol; STM, Streptomycin; FLQ, Fluoroquinolones; SLID, Second-line Injectable anti-tuberculosis drugs
